# Supplementary material for: Drought Stress Tolerance and Photosynthetic Activity of Alloplasmic Lines T. dicoccum x T. aestivum
Source: Int J Mol Sci. 2020 May 9;21(9):3356. doi: 10.3390/ijms21093356 (PMC7246993; doi:10.3390/ijms21093356)
Supplement: Supplementary file 1 [file ijms-21-03356-s001.pdf]

International Journal of *Molecular Sciences*

MDPI

Supplementary Table S1. Characterization of morphological features in plants of wheat species, varieties and alloplasmic lines derived from interspecific crossing *T. dicoccum* Schuebl. var. Araratum (Host.) x *T. aestivum* (Mironovskaya 808). Selection on economically valuable signs was starting with F_3BC_2.

Spesies, Allolines	Height	Stem Characters	Gglume Characters	Kernel Characters	Threshing	Grain Formation
T. dicoccum Schuebl. var. araratum (Host.) from IBBP collection	≤ 140 cm	Brown, medium thickness, under the spike is hollow	Brown, with pubescence; Keel beak short, acute; Sholder shape: narrow, square	Dark red, harder texture	difficult	65%
T. aestivum (M808) from IBBP collection	≤ 130 cm	White, medium thickness, under the spike is hollow	White, without pubescence; Sholder shape: square, slightly slanted in the lower part and raised in the upper part of the spike.	Dark red, vitreous, smooth endosperm	easy	57%
D-a-05	≤ 100 cm	White, medium thickness, under the spike is hollow	White, without pubescence; Keel beak: acuminate; Sholder shape: narrow, square	Dark red, vitreous, smooth endosperm	easy	81%
D-b-05	≤ 100 cm	White, medium thickness, under the spike is hollow	White, without pubescence; Keel beak: acuminate; Sholder shape: narrow, square	Dark red, vitreous, smooth endosperm	easy	78%
D-d-05	≤ 110 cm	White, medium thickness, under the spike is hollow	White, foundation of spikes without pubescence or with pubescence; Keel beak: small, obtuse; Sholder shape: narrow, square	Dark red, vitreous, smooth endosperm	easy	57%
D-d-05b	≤ 110 cm	White, thick, under the spike is hollow	White, without pubescence; Keel beak: acute, long (1 sm); Sholder shape: elevated	Dark red, vitreous, smooth endosperm	easy	70%
D-f-05	≤ 130 cm	White, thick, under the spike is hollow	White, with pubescence (or unevenly brown, with pubescence), Keel beak: acuminate; Sholder shape: wide, square	Dark red, vitreous, smooth endosperm	easy	47%
D-n-05	≤ 130 cm	White, medium thickness, under the spike is hollow	White, with low pubescence; Keel beak: small, obtuse; Sholder shape: wide, square	Dark red, vitreous, smooth endosperm	easy	58%
D-40-05	≤ 117 cm	White, medium thickness, under the spike is hollow	White, without pubescence; Keel beak: small, obtuse; Sholder shape: wide, square	Dark red, vitreous, smooth endosperm	easy	56%
D-41-05	≤ 123 cm	White, medium thickness, under the spike is hollow	White, without pubescence; Keel beak: small, obtuse; Sholder shape: wide, square	Dark red, vitreous, smooth endosperm	easy	59%
D-42-05	≤ 120 cm	White, medium thickness, under the spike is hollow	White, without pubescence; Keel beak: small, obtuse; Sholder shape: wide, square	Dark red, vitreous, smooth endosperm	easy	57%

International Journal of *Molecular Sciences*

MDPI

Supplementary Table S2. Length of PCR fragments detected using microsatellite markers at alloplasmic lines and their parents. ((-) -No PCR fragment).

Marker/Chromosome	T. dicoccum	Mironovskaya 808	D-a-05	D-b-05	D-d-05	D-d-05-b	D-f-05	D-n-05	D-40-05	D-41-05	D-42-05
Xgwm437<br>7D	-	101	101	101	101	101	101	115	101	-	101
Xgwm357<br>1A	127	121	121	121	119	119	127	123	121	121	121
Xgwm3<br>3D	78	78	78	78	78	78	78	78	78	78	78
Xgwm155<br>3A	139	147	147	147	141, 147	141, 147	139	143	147	141	147
Xtaglgap<br>1B	573	569, 573	569, 573	569, 573	569, 573	569, 573	573	569, 573	569, 573	-	569, 573
Xgwm389<br>3B	147	137	137	137	137	137	137	135	137	137	137
Xgwm261<br>2D	154	182	182	182	182	182	182	182	182	182	182
Xgwm513<br>4B	145	-	139, 145	139, 145	139,145	139, 145	139, 147	139, 145	137, 143	137, 143	137, 143
Xgwm190<br>5D	209	217	217	217	217	217	217	217	209, 217	217	217
Xgwm18<br>1B	217	193	193	193	193	193	193	193	193	193	193
Xgwm95<br>2A	121	121	123	123	123	123	123	125	121	121	-
Xgwm160<br>4A	194	184	184	184	184	184	184	180, 190	180, 186	184	184
Xgwm186<br>5A	134	136	136	136	136	136	136	136	136	136	136
Xgwm334<br>6A	122	124	124	124	124	124	124	126	124	-	124
Xgpw2255<br>1D	232	222	222	220	220	224	216	220	-	212	224
Xgwm130<br>7A	122	124	124	124	124	128	124	126	124	128	124
Xgwm192<br>4A,**4B**,4D	196	148, 212	148, 212	148, 212	148, 212	148, 210	148, 196	148, 206	-	148, 212	148, 212

Xgwm148<br>2B	111	115	115	111, 115	111, 115	115	115	115	-	111, 115	115
Xgwm613<br>6B	218	218	218	218	218	218	218	218	218	218	218

International Journal of *Molecular Sciences*

MDPI

```
DN05        GTATTAAGGTTCTTCTCTTCCAGCGTTTAGTATTCAAGTTCTTCTCTTCCAGCCCCCCGG     60
AP013106    GTATTAAGGTTCTTCTCTTCCAGCGTTTAGTATTCAAGTTCTTCTCTTCCAGCCCCCCGG     60
X56186      GTATTAAGGTTCTTCTCTTCCAGCGTTTAGTATTCAAGTTCTTCTCTTCCAGCCCCCCGG     60
GU985444    GTATTAAGGTTCTTCTCTTCCAGCGTTTAGTATTCAAGTTCTTCTCTTCCAGCCCCCCGG     60
            ************************************************************

DN05        CCCCTCTTTGATAAGGAAAGTTTGCATTTCTCAAATAAAAAATGACAAATATGGTTCGAT     120
AP013106    CCCCTCTTTGATAAGGAAAGTTTGCATTTCTCAAATAAAAAATGACAAATATGGTTCGAT     120
X56186      CCCCTCTTTGATAAGGAAAGTTTGCATTTCTCAAATAAAAAATGACAAATATGGTTCGAT     120
GU985444    CCCCTCTTTGATAAGGAAAGTTTGCATTTCTCAAATACAAAATGACAAATATGGTTCGAT     120
            ************************************* **********************

DN05        GGCTCTTCTCCACTAGCAGGTTTACTGCTTTCTATTTGCACTTTTGTATTAAGTTTCCTT     180
AP013106    GGCTCTTCTCCACTAGCAGGTTTACTGCTTTCTATTTGCACTTTTGTATTAAGTTTCCTT     180
X56186      GGCTCTTCTCCACTAGCAGGTTTACTGCTTTCTATTTGCACTTTTGTATTAAGTTTCCTT     180
GU985444    GGCTCTTCTCCACTAGCAGGTTTACTGCTTTCTATTTGCACTTTTGTATTAAGTTTCCTT     180
            ************************************************************

DN05        ATATATACGATTTTTTATTATTTTCTATTTGTCTATTTTTCTTTTTAGTGCGTTTTATTT     240
AP013106    ATATATACGATTTTTTATTATTTTCTATTTGTCTATTTTTCTTTTTAGTGCGTTTTATTT     240
X56186      ATATATACGATTTTTTATTATTTTCTATTTGTCTATTTTTCTTTTTAGTGCGTTTTATTT     240
GU985444    ATATATACGATTTTATATTATTTTCTATTTGTCTATTTTTATTTATTTGTCGTTTTATTT     240
            ************** ************************* *** *     **********

DN05        CGATTATTCTTCTCCCAATTTGCAATCTTTTCGGAGCCTCCTTCATTATTACTCTTCCTC     300
AP013106    CGATTATTCTTCTCCCAATTTGCAATCTTTTCGGAGCCTCCTTCATTATTACTCTTCCTC     300
X56186      CGATTATTCTTCTCCCAATTTGCAATCTTTTCGGAGCCTCCTTCATTATTACTCTTCCTC     300
GU985444    ATATTATTCTTCTCCCAATTTTACATCTTTTCGGAGCCTCCTTCCTTATTACTCTTCCTC     300
              *******************   ******************** ***************

DN05        CAGAGATTCAGGATCCCCAAGCTCTAGCTCATTTAGCAGGGCTAAACTTCTATCTGAGCC     360
AP013106    CAGAGATTCAGGATCCCCAAGCTCTAGCTCATTTAGCAGGGCTAAACTTCTATCTGAGCC     360
X56186      CAGAGATTCAGGATCCCCAAGCTCTAGCTCATTTAGCAGGGCTAAACTTCTATCTGAGCC     360
GU985444    CAGAGATTCAGGATCCCCAAGCTCTAGCTCATTTAGCAGGGCTAAACTTCTATCTGAGCC     360
            ************************************************************

DN05        TTTACGAGCAGGATC   375
AP013106    TTTACGAGCAGGATC   375
X56186      TTTACGAGCAGGATC   375
GU985444    TTTACGAGCAGGATC   375
            ***************
```

Supplementary Figure S1. Alignment of the *orf256* sequences of mtDNA, including those from D-N-05 alloplasmic line (sequenced in this work), *T. timopheevii* (AP013106), *T. timopheevii* x *T. aestivum* CMS line (X56186) and *T. aestivum* (GU985444).

```
                    rps19f
            ------------------------>
AP013106    TGCTCCGTACTCATTTACAATGGAAAAACTCCTGTTCGTTGTAAGATCACTGAAGGAAAG     60
X56186      TGCTCCGTACTCATTTACAATGGAAAAACTCCTGTTCGTTGTAAGATCACTGAAGGAAAG     60
GU985444    TGCTCCGTACTCATTTACAATGGAAAAACTCCTGTTCGTTGTAAGATCACTGAAGG----     56
            ********************************************************
                                                  rps19r
                                      <----------------------
AP013106    GTTGGTCATAAATTTGGAGAGTTTGCTTTTACACGGAGACGAAGACCCTAT     111
X56186      GTTGGTCATAAATTTGGAGAGTTTGCTTTTACACGGAGACGAAGACCCTAT     111
GU985444    -----TCATAAATTTGGAGAGTTTGCTTTTACACGGAGACGAAGACCCTAT     102
                 **********************************************
```

Supplementary Figure S2. Alignment of the *rps-19-p* sequences of mtDNA. The samples corresponding to Acc. No are presented in the legend to Suppl.Fig.1. The primers used for PCR are indicated by arrows. No sequences were obtained in this work because of a short length of the PCR fragment.

```
DQ195069    TGTTTTAGATTTATACGACTTTTTTTTCTCTGAGAAAGATTTATATGACTCTGACTGCTT    60
Tdic        TGTTTTAGATTTATACGACTTTTTTTTCTCTGAGAAAGATTTATATGACTCTGACTGCTT    60
DF05        TGTTTTAGATTTATACGACTTTTTTTTCTCTGAGAAAGATTTATATGACTCTGACTGCTT    60
D4105       TGTTTTAGATTTATACGACTTTTTTTTCTCTGAGAAAGATTTATATGACTCTGACTGCTT    60
            ************************************************************
                                                     P40
DQ195069    ATGTTTTTTGTTTCAACGTGGTTTCACCTTGTGATATGGATTGCCTTGATGACCAGGAAG    120
Tdic        ATGTTTTTTGTTTCAACGTGGTTTCACCTTGTGATATGGATTGCCTTGATGAACAGGAAG    120
DF05        ATGTTTTTTGTTTCAACGTGGTTTCACCTTGTGATATGGATTGCCTTGATGAACAGGAAG    120
D4105       ATGTTTTTTGTTTCAACGTGGTTTCACCTTGTGATATGGATTGCCTTGATGAACAGGAAG    120
            **************************************************** *******

DQ195069    AAGAAAGTGCGCAGGAGAAGCACTGGTCCTGATTCGGTTGCTGAAACCATCAAGAAGTGG    180
Tdic        AAGAAAGTGCGCAGGAGAAGCACTGGTCCTGATTCGGTTGCTGAAACCATCAAGAAGTGG    180
DF05        AAGAAAGTGCGCAGGAGAAGCACTGGTCCTGATTCGGTTGCTGAAACCATCAAGAAGTGG    180
D4105       AAGAAAGTGCGCAGGAGAAGCACTGGTCCTGATTCGGTTGCTGAAACCATCAAGAAGTGG    180
            ************************************************************
```

Supplementary Figure S3. Alignment of a part of the *Dreb-B1* sequences, isolated from *T. dicoccum* and alloplasmic lines D-41-05 and D-f-05. The corresponding sequence of *T. aestivum* was downloaded from NCBI database under the Acc. No DQ195069. The position of P40 primer used for AS-PCR is indicated. The characteristic SNP is localized at the 3′-end of the primer (see text). The start ATG-codon of translation is depicted by the vertical line with arrow.
